# COVID-19 vaccine-related misinformation identification among Chinese residents during a regional outbreak

**DOI:** 10.3389/fpubh.2023.1258466

**Published:** 2023-10-06

**Authors:** Jie Li, Yueying Chen, Xiaoquan Zhao, Xiaobing Yang, Fan Wang

**Affiliations:** ^1^School of Journalism and Communication/National Media and Experimental Teaching Center, Jinan University, Guangzhou, China; ^2^College of Media and International Culture, Zhejiang University, Hangzhou, China; ^3^Department of Communication, George Mason University, Fairfax, VA, United States; ^4^School of Journalism and Communication, Jinan University, Guangzhou, China; ^5^Fudan Development Institute, Fudan University, Shanghai, China

**Keywords:** COVID-19 vaccine, misinformation identification, information source usage, information source trust, perceived information quality

## Abstract

**Objectives:**

Misinformation about the COVID vaccines poses a significant challenge to vaccination efforts in many countries. This study examined Chinese citizens’ ability to correctly identify COVID-19 vaccine misinformation in geographic areas with and without a regional outbreak. We also investigated the associations between misinformation identification and information source usage, source trust, perceived information quality, and demographic characteristics.

**Setting:**

The online survey was conducted in four cities from June 8th to 15th, 2021 in Guangdong Province, two of which were experiencing a regional surge of COVID-19 delta variant infections, and four cities in Hunan Province, a neighboring province largely unaffected.

**Participants:**

A total of 4,479 individuals aged 18 and above completed the online questionnaire. Given survey length, those who finished the study under 5 min were excluded, resulting in a final sample of 3,800.

**Outcome measurements:**

Misinformation identification, source exposure, source trust, and perceived information quality.

**Results:**

Results showed slightly higher levels of correct misinformation identification in surge vs. non-surge areas. Trust in official information sources was positively associated with correct misinformation identification in full sample analysis, while trust in informal sources was negatively associated with the same outcome. Perceived information quality was positively associated with correct misinformation identification in the full sample.

**Conclusion:**

Information providers in China should enhance the quality of the vaccine information they provide, and the Chinese public should balance their usage of different sources of information to acquire vaccine knowledge.

## 1. Introduction

In May 2021, a large-scale outbreak caused by the coronavirus variant, Delta, happened in Guangzhou (1). This regional outbreak urged the Chinese government to speed up nationwide vaccination to better protect its population against the COVID-19 virus. However, China’s COVID vaccines had only become widely available to the general public for about 3 months by that time (2). China’s COVID control efforts were heavily focused on the promotion of daily preventative behaviors such as wearing masks, washing hands, and social distancing (3). There was a general lack of information about the vaccines, which left the door open for the growth and influence of misinformation (4–6). In China, in addition to official information sources, such as government-owned news outlets, informal sources such as interpersonal networks and social media also play an important role as information purveyors. Large amounts of misinformation and conspiracy theories exist in these sources (7–9). Heavy use of social media and other informal sources often leads to exposure to misinformation, which in turn may inflate risk perceptions about the vaccines, resulting in more negative attitudes toward vaccination (10, 11).

What kinds of misinformation or misunderstanding about the COVID-19 vaccines might have existed during the Guangzhou regional outbreak? How well were people able to identify misinformation when they saw it? Were there any differences in people’s ability to identify vaccine-related misinformation between the surge areas (i.e., areas directly affected by the outbreak) and non-surge areas? How was misinformation identification associated with information source usage, trust in these sources, perceived information quality, and individual characteristics? This study aims to address these questions through a survey conducted during the outbreak of local residents from eight Chinese cities in two provinces in China.

## 2. Background

### 2.1. Misinformation

Health misinformation is defined as “a health-related claim of fact that is currently false due to a lack of scientific evidence” (12). Vaccine misinformation is mostly anti-vaccine in nature (13) and tends to arouse public fear and decrease vaccine confidence (14). Typical contents of vaccine misinformation include false vaccine safety and effectiveness claims, inaccurate information about vaccination procedures, conspiracy theories, and so on (15). Previous research about HPV, MMR, and other vaccines has generated ample evidence for the negative effects of misinformation on public risk perceptions, attitudes, and behaviors (16, 17). As COVID-19 swept through the world, misinformation regarding its causes, treatments, and mechanisms of spread has surged so much that the WHO declared COVID-19 an “infodemic” (18). A cross-national study of multiple countries, including China, showed vaccine-related misinformation to be a major theme of the COVID-19 infodemic (11). For example, misinformation against COVID-19 vaccines may increase confusion and hesitation concerning types of vaccines. The COVID-19 vaccines available to the Chinese public in 2021 were inactivated vaccines, which differ from other vaccines, such as the mRNA vaccines. False messages may impede individuals from taking necessary prevention by describing inactivated vaccines as totally ineffective. A growing body of literature has documented the deleterious effects of misinformation on vaccine-related attitudes and behaviors (19, 20). To effectively promote COVID-19 vaccines in China, it is important to know what types of misinformation are prevalent among the Chinese public and how well people in China are able to differentiate misinformation from accurate information about the vaccines.

Although COVID-19 is considered a national health crisis, severity of the situation may vary in different areas, partly due to China’s strict health policies that have prevented regional outbreaks from spreading across cities or provinces. In this study, surge areas refer to places where a COVID-19 outbreak is currently occurring, while non-surge areas mean regions with relatively few or no confirmed cases of COVID-19. During a regional outbreak, people living in affected and unaffected areas face vastly different life circumstances and may hold different vaccine-related beliefs and perceptions. Prior studies indicated that the information demands and behaviors of the public may change during a crisis. For instance, individuals amid health emergencies might want more information to stay informed of the ever-changing situation (21). But the urgency to regulate negative emotions such as anxiety and fear could also lead them to neglect information quality (22), making them vulnerable to misinformation. Moreover, the content, type, and framing of information are likely to be different between surge and non-surge areas. Non-surge areas often tend to focus on the promotion of daily precautionary measures, whereas information in surge regions may more often adopt a crisis news framing or try to shift public attention during recovery (23, 24). With this in mind, this study intends to see if individuals’ ability to identify vaccine-related misinformation would differ between surge and non-surge areas.

*RQ1*: How well could Chinese residents identify misinformation about the COVID-19 vaccines during the Guangzhou regional outbreak?

*RQ2*: Did the ability to identify vaccine-related misinformation differ between residents from surge and non-surge areas?

### 2.2. Information sources, trust, and perceived quality

In many ways, people’s information sources can shape what they see and what they believe (25, 26). When it comes to vaccination, previous research revealed that the sources from which individuals obtained information played a crucial role in their vaccination attitudes. For instance, prior studies demonstrated that individuals exposed to traditional news sources took the disease more seriously and expressed stronger pro-vaccine attitudes, while individuals predominately depending on the Internet tended to show less confidence in vaccine safety and effectiveness (27–29). A study conducted in China in the early days of the pandemic showed that the more diverse the channels people use, the greater one’s likelihood to hold correct perceptions, and the greater one’s ability to identify misleading information (30). Exploring how individuals use and feel about different information sources can thus inform the understanding of their perceptions and attitude toward vaccines (31, 32).

Health information can be obtained from many different sources, ranging from news organizations, social media, health professionals to interpersonal networks. Past research categorized sources of health information into formal and informal sources (33). Formal sources refer to those whose credibility is endorsed by health departments and professionals, for instance, government websites and health resources (34, 35). These sources are usually more reliable and trustworthy than non-official social media outlets and word of mouth. For example, research found that those who consulted physicians as the primary source of vaccine-related information had better knowledge and more positive vaccine attitudes (36, 37). Informal sources refer to non-governmental, alternative outlets, such as general social media sites and online search engines (33). Among all the information sources, social media appear to have carried the most misinformation about COVID-19 and COVID-19 vaccines (38–40), mostly from informal sources. Besides, there is also evidence that false information tends to travel faster and broader on social media (41, 42). The abundance of health information on social media has made it difficult for the public to verify information accuracy, impeding effective public health response (8, 43).

Based on existing literature, this study adopts the dichotomous categorization of information sources, namely official versus informal sources. This dichotomy is particularly relevant for the China context, where official channels are quickly established and tightly controlled by the government whenever a health emergency occurs. Moreover, in China, professional news agencies have stayed highly consistent with the government to deliver scientific and timely updates on COVID-19, due to the supervision of the government on news coverage during health emergencies (44). Hence, we include news organizations as official sources in this study. We are interested in learning whether the use of these two categories of information sources would show different patterns of associations with the public’s ability to identify vaccine-related misinformation.

It should be noted that accessing information does not necessarily mean accepting it. Individuals’ perceptions of information sources play a role in their impact. Trust in information sources is considered an essential precondition for information acquisition and positive responses toward the health advice offered by the relevant sources (45). Recent studies on COVID-19 found that trust in information sources positively predicts the adoption of protective behaviors and favorable vaccination attitudes (25, 46). On the other hand, lower trust in scientific institutions and government was found to be positively associated with misinformation beliefs in a recent longitudinal survey study (47).

In addition to trust, perceived quality of health information is also an important factor in information consumption and impact, particularly in the E-health era (48–50). Recent research has examined the quality of COVID-19 information (50–52). For example, Halboub et al. (50) assessed the quality and readability of web-based Arabic health information on COVID-19. Stern et al. (52) investigated the quality of web-based information about preventive measures and self-care methods at the beginning of the COVID-19 pandemic. While highlighting the importance of information quality, these studies were based on content analysis and did not explore the perceptions of information receivers.

In this study, we assess Chinese residents’ use of different information sources, their trust in these sources, and their general perception of the quality of the information they have been receiving about the COVID-19 vaccines. The associations between these informational variables and the public’s ability to identify vaccine-related misinformation constitutes another key interest of this research.

*H1*: (a) Exposure to official sources is positively associated with the ability to identify misinformation, while (b) exposure to informal sources is negatively associated with the ability to identify misinformation.

*H2*: (a) Trust in official sources is positively associated with the ability to identify misinformation, while (b) trust in informal sources is negatively associated with the ability to identify misinformation.

*H3*: Perceived information quality is positively associated with the ability to identify misinformation.

### 2.3. Individual background factors

Past research showed that sociodemographic factors were significant predictors of vaccine knowledge and/or misinformation beliefs (36, 53, 54). For example, one study found that older adults people exposed to erroneous information regarding vaccines and COVID-19 in the media were more likely to hold misperceptions (55). Another study found that while income was unrelated to misinformation exposure, it was negatively associated with misinformation acceptance (56). Given that COVID-19 represents a particularly grave danger for those with preexisting conditions, individual health status might also have a role to play in people’s ability to identify vaccine-related misinformation. Our last research question, therefore, investigates the relationships between misinformation identification and individual’s sociodemographic background and general health status.

*RQ3*: Was misinformation identification associated with sociodemographic factors and individual health status?

## 3. Methods

### 3.1. Study design and setting

A cross-sectional online survey was conducted in Guangdong Province and Hunan Province from June 8th to 15th, 2021. During this period, Guangdong was the center of the severe outbreak caused by the Delta variant, which clustered in the southern coastal area. Hunan, although adjacent to Guangdong to the north, was not affected. Four cities were chosen within each province to represent different levels of economic development. In Guangdong Province, Guangzhou, Shantou, Maoming, and Meizhou were selected. In Hunan Province, Changsha, Changde, Chenzhou, and Huaihua were selected. Guangzhou and Maoming were categorized as surge areas and the rest of the cities non-surge areas.

The survey was distributed through wjx.cn, the largest online survey platform in China with a demonstrated record of generating high-quality survey data (36, 57). Ethics approval for the current study was obtained from the Social Science Ethics Committee of Jinan University.

### 3.2. Participants

Snowballing was a key mechanism in both types of recruitment. Given strict COVID regulations, data collection for this study took the form of an online questionnaire. Since the development level of different cities in China varies greatly, different strategies were used for recruitment and data collection.

Guangzhou and Changsha are provincial capital cities with large populations and relatively mature community organizations. Researchers used WeChat, the leading social media platform in China, to recruit participants. Each city was divided into two strata, urban and suburban. Within each stratum, four residential communities were selected, each having WeChat groups with high coverage of the community membership. Recruitment materials were posted in the WeChat groups, and those who agreed to take the survey could fill out the questionnaire either on their mobile phones or using their personal computers. Both Guangzhou and Changsha are home to many major universities, where the vast majorities of students live in dormitories on campus. To supplement the local sample, researchers also recruited college students through their WeChat groups.

In the other six relatively underdeveloped cities, community-based WeChat groups were less popular and unlikely to provide satisfactory coverage of the resident populations. In these cities, a two-prong strategy was used for recruitment. For younger working residents, we sent study invitations to WeChat groups of different businesses and work units. For older adults residents, we recruited local community volunteers to directly approach and invite older adults members of their communities to fill out the questionnaire.

A total of 4,479 individuals completed the questionnaire. Only residents aged 18 and above were allowed to participate in the study. Given survey length, those who finished the study under 5 min were excluded, resulting in a final sample of 3,800 (84.8% of the total finished sample).

### 3.3. Measures

#### 3.3.1. Misinformation identification

Ten false vaccine-related statements were presented, and participants were asked to indicate each as true or false. All statements were extracted from authoritative health information platforms, including China Central Television (CCTV) News, one professional health consultation website (dxy.cn, akin to WebMD in the U.S.), and one major online rumor-busting platform in China.^1^ While most of the statements were existing rumors and false information, a few were converted from factual information to expand the coverage of the misinformation test. In order to understand respondents’ overall capacity to recognize misinformation, the statements concerned diverse aspects, including necessity (2 items), effectiveness (2 items), benefits (1 item), safety (2 items), procedure (1 item), and precautions (2 items) related to vaccination against COVID-19. Correct responses (1 point each) were summed into a total score representing participants’ ability to identify vaccine-related misinformation.

#### 3.3.2. Sources of COVID-19 vaccine information

Participants were asked about the frequency with which they were exposed to information about COVID-19 vaccines from nine different sources. Responses were indicated on a 5-point scale (1 = “never” to 5 = “frequently”). These nine sources were combined into two categories. One represented official sources with varying degrees of affiliation with the government, including traditional media, news media websites or apps, work unit/school, health resources, and community administrative agencies. The other was a group of informal sources, including online search engines, social media, short video platforms, and interpersonal sources. A usage index for each category was created by averaging the appropriate items.

#### 3.3.3. Trust in COVID-19 vaccine information sources

On a 5-point scale (1 = “do not trust at all” to 5 = “deeply trust”), participants reported the level of trust they had in the same nine sources as in the previous measure. Similarly, these nine items were combined through averaging into two categories, one reflecting trust in official sources, the other trust in informal sources.

#### 3.3.4. Perceived quality of COVID-19 vaccine information

Four items were adapted from Lee et al. (58) to assess perceived quality of the COVID-19 vaccine information participants had received, regardless of sources. The items tapped into information credibility, clarity, relevance, and timeliness (e.g., “The COVID-19 vaccination information I got is trustworthy”). A 5-point scale (1 = “strongly disagree” to 5 = “strongly agree”) was used to record responses. A summary score was created by averaging across items.

#### 3.3.5. Demographics and health status

Participants’ gender, age, education, and income were measured following norms in demography research in China. Self-reported health status was measured on a 4-point scale: “excellent,” “good,” “fair,” and “poor.” The last two categories were later combined due to small group sizes, resulting in a three-level measure.

### 3.4. Analysis

Pearson chi-square and independent-sample t-test were used to examine the differences between participants from surge and non-surge areas in sample characteristics, misinformation identification, source exposure, source trust, and perceived information quality. A series of logistic regressions were conducted to predict correct identification of each misinformation statement based on source exposure, sources trust, perceived information quality, and demographic factors. The same set of covariates was also used in an ordinary least squares (59) regression to predict the total score of correct misinformation identification across the ten statements. All analyses were performed using SPSS v.28 (IBM).

## 4. Results

### 4.1. Sample characteristics

Sample characteristics are presented in Table 1. Of the final sample (*N* = 3,800), 1,386 (36.5%) were from surge areas, 2,414 (63.5%) were from non-surge areas. The sample was diverse in sociodemographic characteristics. Most participants were female (63.5%) and 85.4% of them were under 50 in age. More than half of the participants (53.6%) had a college degree or above. Most participants (83.2%) earned 10,000 yuan or less per month. The majority of the sample rated their current health as excellent (72.2%), while very few rated their health as fair or poor (7.0%). Compared to those from non-surge areas, participants from surge areas were slightly younger (*p* < 0.001), better educated (*p* < 0.001), earning a higher income (*p* < 0.001), and seeing themselves as in better health (*p* < 0.001). There was no difference between the two subsamples in terms of gender composition (*p* = 0.742).

**Table 1:** Sample characteristics.

	Entire sample (*N* = 3,800) %	Surge areas (*N* = 1,386) %	Non-surge areas (*N* = 2,414) %	*p* ^a^
**Gender**				0.742
Male	36.5%	36.2%	36.7%	
Female	63.5%	63.8%	63.3%	
**Age**				<0.001
18–29	25.7%	23.7%	27.3%	
30–39	30.4%	35.2%	26.6%	
40–49	29.3%	27.0%	31.2%	
50^+^	14.6%	14.2%	14.9%	
**Education level**				<0.001
High school or less	23.6%	17.4%	28.4%	
Associate degree	22.8%	21.5%	23.8%	
College graduate	46.9%	53.0%	42.1%	
Postgraduate	6.7%	8.1%	5.7%	
**Monthly income**				<0.001
¥0–1,000	13.9%	13.2%	14.4%	
¥1,001–5,000	36.0%	27.3%	42.8%	
¥5,001–10,000	33.3%	33.0%	33.6%	
¥10,001^+^	16.8%	26.6%	9.2%	
**Health status**				<0.001
Excellent	72.2%	77.2%	68.3%	
Good	20.8%	17.8%	23.2%	
Fair or poor	7.0%	5.0%	8.5%	

a Pearson chi-square test between surge and non-surge areas.

### 4.2. Ability to identify misinformation

To answer RQ1, Table 2 presents the full sample’s overall performance on misinformation identification, which showed reasonable competence. The mean score for the entire sample was 7.84 (SD = 1.69), meaning that, on average, participants were able to correctly identify about 8 out of the 10 misinformation statements. Specific to individual items, the rate of correct identification was excellent for items 2, 4, 5 and 6 (all above 90.0%), while relatively poor for item 3 (57.0%) and item 9 (38.3%).

**Table 2:** Misinformation identification.

Items	Entire sample (*N =* 3,800) %	Surge areas(*N =* 1,386) %	Non-surge areas(*N =* 2,414) %	*p* ^a^
(1) There is no need to get vaccinated as long as I take precautionary measures like wearing masks, washing hands, and keeping social distance	72.6	72.2	72.8	0.710
(2) I do not need to get vaccinated now that most people around me have been vaccinated	96.1	96.3	95.9	0.502
(3) The domestic vaccine is an inactivated virus vaccine with a period of protection of only half a year	57.0	58.3	56.1	0.176
(4) I do not need to wear masks anymore after vaccination	95.7	97.4	94.3	<0.001
(5) The COVID-19 vaccine is no longer effective now that the COVID-19 virus has mutated	96.0	96.0	96.0	0.984
(6) Getting COVID-19 vaccine often causes severe adverse reactions	92.3	92.9	91.9	0.267
(7) People aged 60 and above are not suitable for COVID-19 vaccination	73.5	73.9	73.2	0.629
(8) I can get the second dose of COVID 19 vaccine 10 days after the first dose	89.7	91.7	88.1	<0.001
(9) Patients with chronic diseases such as hypertension and diabetes are not advised to take COVID-19 vaccine	38.3	39.1	37.6	0.354
(10) Xiaoming was bitten by a dog 3 days after receiving his first dose of COVID 19 vaccine, and should not be vaccinated against rabies for the time being	72.7	73.8	71.9	0.201
Mean # of correct responses (60)	7.84 (1.69)	7.92 (1.66)	7.78 (1.70)	0.012

a Pearson chi-square test between surge and non-surge areas.

### 4.3. Differences between surge and non-surge areas

#### 4.3.1. Differences in misinformation identification

Results answering RQ2 are also presented in Table 2. As shown, there was a significant difference in the mean scores for misinformation identification between surge areas and non-surge areas (*M* = 7.92 vs. 7.78, *p* = 0.012). For individual items, significant differences were observed for item 4 and item 8. For item 4, the rate of correct identification was 97.4% for surge areas and 94.3% for non-surge area (*p* < 0.001). For item 8, the rate of correct identification was 97.7% for surge areas and 88.1% for non-surge areas (*p* < 0.001). No significant difference was found for the other items.

#### 4.3.2. Differences in informational variables

Table 3 presents descriptive statistics on exposure to different sources of COVID vaccine information, trust in these sources, and perceived quality of the vaccine information received for the full sample. It also shows the differences between surge and non-surge areas. As indicated in Table 3, participants from surge and non-surge areas reported similar levels of exposure to COVID vaccine information from official (*M* = 3.63 vs. 3.65, *p* = 0.457) and informal sources (*M* = 3.84 vs. 3.84, *p* = 0.816). They also had similar levels of trust in official sources (*M* = 4.20 vs. 4.18, *p* = 0.350) and perceived information quality (*M* = 4.26 vs. 4.32, *p* = 0.063). Participants from surge areas reported slightly lower levels of trust in informal sources (*M* = 3.61 vs. 3.66, *p* = 0.048), the only difference that reached statistical significance among the information variables.

**Table 3:** Source exposure, source trust, and perceived information quality.

	Entire sample (*N =* 3,800)	Surge areas (*N =* 1,386)	Non-surge areas (*N =* 2,414)	*p* ^a^	Alpha
	Mean (60)	Mean (60)	Mean (60)		
Exposure to official sources^b^	3.64 (0.90)	3.63 (0.89)	3.65 (0.91)	0.457	0.85
Exposure to informal sources^c^	3.84 (0.81)	3.84 (0.80)	3.84 (0.82)	0.816	0.77
Trust in official sources^b^	4.19 (0.70)	4.20 (0.69)	4.18 (0.71)	0.350	0.92
Trust in informal sources^c^	3.63 (0.83)	3.61 (0.83)	3.66 (0.82)	0.048	0.89
Perceived information quality	4.29 (0.80)	4.26 (0.80)	4.32 (0.79)	0.063	0.97

a Independent sample *t*-test between surge and non-surge areas.

b Official sources: traditional media, news media sites or apps, work unit/school, health resources and community.

c Informal sources: search engines, social media, short video platforms and interpersonal sources.

### 4.4. Correlates of misinformation identification

#### 4.4.1. Individual misinformation items

H1–H3 and RQ3 asked about factors associated with the respondents’ ability to identify COVID-19 vaccine misinformation. Table 4 presents the logistic regressions examining the relationships between correct identification of each misinformation statement and demographics, health status, source usage, source trust, and perceived information quality. To further investigate RQ2, place of residence (surge vs. non-surge areas) was also included in the models. Results showed complex relationships between sociodemographic factors and the ability to identify specific misinformation items. For example, those who answered item 3 correctly were less likely to be 30 or older, more likely to have higher education levels, less likely to report good (vs. excellent) health, and less likely to report a monthly income of ¥1,001–5,000 (vs. ¥0–1,000). Those who answered item 9 correctly were more likely to be female, aged 50^+^ (vs. 18–29), and less likely to have a monthly income of ¥1,001–5,000 (vs. ¥0–1,000), and more likely to report fair or poor (vs. excellent) health condition. And those who answered item 7 correctly were more likely to be 30 or older and hold a postgraduate degree (vs. high school or less).

**Table 4:** Logistic regression predicting correct identification of individual misinformation items.

	Item1		Item 2		Item 3		Item 4		Item 5		Item 6		Item 7		Item 8		Item 9		Item 10	
	OR	*p*	OR	*p*	OR	*p*	OR	*p*	OR	*p*	OR	*p*	OR	*p*	OR	*p*	OR	*p*	OR	*p*
**Gender**																				
Male	Ref.		Ref.		Ref.		Ref.		Ref.		Ref.		Ref.		Ref.		Ref.		Ref.	
Female	1.215	0.013	1.339	0.097	1.005	0.941	1.582	0.006	1.118	0.533	1.154	0.280	1.112	0.185	1.154	0.212	1.222	0.006	1.073	0.372
**Age**																				
18–29	Ref.		Ref.		Ref.		Ref.		Ref.		Ref.		Ref.		Ref.		Ref.		Ref.	
30–39	1.057	0.605	0.934	0.793	0.816	0.038	0.641	0.088	0.619	0.096	0.804	0.251	1.512	<0.001	0.962	0.812	0.888	0.237	1.114	0.327
40–49	1.226	0.066	0.896	0.676	0.699	<0.001	0.578	0.035	0.475	0.009	0.805	0.262	1.707	<0.001	0.914	0.579	1.022	0.833	0.907	0.379
50^+^	1.040	0.767	0.585	0.059	0.791	0.052	0.498	0.017	0.352	<0.001	0.644	0.041	2.162	<0.001	0.845	0.377	1.475	0.001	0.709	0.008
**Education level**																				
High school or less	Ref.		Ref.		Ref.		Ref.		Ref.		Ref.		Ref.		Ref.		Ref.		Ref.	
Associate degree	1.063	0.574	0.920	0.727	1.267	0.019	1.103	0.667	0.976	0.923	1.540	0.014	1.004	0.975	1.354	0.051	1.192	0.089	1.290	0.021
College graduate	1.295	0.011	1.269	0.312	1.242	0.019	1.327	0.201	0.929	0.759	1.785	<0.001	0.997	0.977	1.470	0.008	1.099	0.325	1.121	0.260
Postgraduate	1.305	0.140	0.845	0.658	1.416	0.030	0.729	0.379	1.356	0.490	1.723	0.079	1.578	0.017	1.567	0.097	1.306	0.100	1.331	0.127
**Monthly income**																				
¥0–1,000	Ref.		Ref.		Ref.		Ref.		Ref.		Ref.		Ref.		Ref.		Ref.		Ref.	
¥1,001–5,000	1.070	0.570	1.391	0.221	0.781	0.028	1.559	0.079	1.119	0.702	1.232	0.274	0.947	0.653	1.248	0.186	0.766	0.019	1.156	0.222
¥5,001–10,000	1.202	0.159	1.480	0.185	0.870	0.251	1.670	0.069	1.453	0.253	1.664	0.020	1.058	0.668	1.387	0.080	0.976	0.841	1.478	0.003
¥10,001^+^	1.200	0.239	1.168	0.652	0.798	0.113	2.094	0.038	0.900	0.770	1.332	0.267	1.061	0.703	1.310	0.232	0.952	0.733	1.608	0.003
**Health status**																				
Excellent	Ref.		Ref.		Ref.		Ref.		Ref.		Ref.		Ref.		Ref.		Ref.		Ref.	
Good	1.092	0.352	0.925	0.710	0.841	0.036	0.864	0.456	0.687	0.056	0.972	0.855	0.968	0.732	1.068	0.627	1.168	0.067	1.230	0.030
Fair or poor	0.985	0.919	1.451	0.330	1.119	0.406	0.946	0.856	0.858	0.628	1.083	0.743	1.204	0.247	1.082	0.713	1.528	0.002	0.918	0.550
**Information-related factors**																				
Exposure to official sources^a^	0.798	<0.001	0.672	0.015	0.956	0.418	0.861	0.307	0.821	0.211	0.847	0.135	1.147	0.029	1.036	0.696	1.118	0.050	0.933	0.273
Exposure to informal sources^b^	0.919	0.245	1.251	0.211	0.970	0.643	1.228	0.214	1.136	0.467	1.076	0.560	0.867	0.052	0.876	0.213	0.945	0.391	1.047	0.531
Trust in official sources^a^	1.390	<0.001	2.075	<0.001	1.185	0.019	1.345	0.105	2.325	<0.001	1.473	0.004	1.186	0.035	1.121	0.329	1.169	0.037	1.404	<0.001
Trust in informal sources^b^	0.970	0.649	0.528	<0.001	0.986	0.817	0.564	<0.001	0.610	0.006	0.710	0.006	0.950	0.460	0.877	0.196	1.059	0.343	0.817	0.003
Perceived information quality	1.134	0.039	1.365	0.018	1.085	0.136	1.372	0.010	1.504	0.001	1.516	<0.001	1.240	<0.001	1.321	<0.001	0.976	0.662	1.051	0.415
Surge areas vs. non-surge areas	0.923	0.299	1.083	0.657	1.059	0.412	2.014	<0.001	0.989	0.953	1.034	0.800	1.012	0.876	1.435	0.002	1.058	0.429	1.000	0.997
Nagekkerke *R* square	0.026		0.050		0.021		0.059		0.087		0.055		0.048		0.027		0.029		0.034	

a Official sources: traditional media, news media sites or apps, work unit/school, health resources and community.

b Informal sources: search engines, social media, short video platforms and interpersonal sources.

As for the information variables, trust in official sources was positively associated with the correct identification of item 1 (OR = 1.390, *p* < 0.001), item 2 (OR = 2.075, *p* < 0.001), item 3 (OR = 1.185, *p* = 0.019), item 5 (OR = 2.325, *p* < 0.001), item 6 (OR = 1.473, *p* = 0.004), item 7 (OR = 1.186, *p* = 0.035), item 9 (OR = 1.169, *p* = 0.037) and item 10 (OR = 1.404, *p* < 0.001). On the other hand, trust in informal sources was negatively associated with the correct identification of item 2 (OR = 0.528, *p* < 0.001), item 4 (OR = 0.564, *p* < 0.001), item 5 (OR = 0.610, *p* = 0.006), item 6 (OR = 0.710, *p* = 0.006) and item 10 (OR = 0.817, *p* = 0.003). Exposure to official sources was positively associated with the correct identification of item 7 (OR = 1.147, *p* = 0.029) and item 9 (OR = 1.118, *p* = 0.050), but negatively associated with the correct identification of item 1 (OR = 0.798, *p* < 0.001) and item 2 (OR = 0.672, *p* = 0.015). Exposure to informal sources was unrelated to any of the outcomes. Moreover, perceived information quality was positively associated with the correct identification of item 1 (OR = 1.134, *p* = 0.039), item 2 (OR = 1.365, *p* = 0.018), item 4 (OR = 1.372, *p* = 0.010), item 5 (OR = 1.504, *p* = 0.001), item 6 (OR = 1.516, *p* < 0.001), item 7 (OR = 1.240, *p* < 0.001), and item 8 (OR = 1.321, *p* < 0.001). Finally, surge area participants performed better on item 4 (OR = 2.014, *p* < 0.001) and item 8 (OR = 1.435, *p* = 0.002) than those from non-surge areas.

#### 4.4.2. Total score

To further examine H1–H3 and RQ3, the same set of predictors shown in Table 4 were also used in an OLS regression to predict the total score of correct responses for the misinformation test. As shown in Table 5, exposure to COVID-19 vaccine information from official sources (*β* = −0.026, *p* = 0.277) and from informal sources (*β* = −0.021, *p* = 0.404) were unrelated to the total score of correct responses. H1 was rejected. Trust in COVID-19 vaccine information from official sources was positively associated with the total score (*β* = 0.141, *p* < 0.001), whereas trust in informal sources was negatively associated with the total score (*β* = −0.061, *p* = 0.010). Both H2a and H2b were supported. Furthermore, perceived quality of COVID-19 vaccine information emerged as a positive predictor (*β* = 0.095, *p* < 0.001). Hence, H3 was supported. Whether participants were from surge areas or non-surge areas was not associated with the total score (*β* = 0.023, *p* = 0.162).

**Table 5:** OLS regression model predicting the total score for misinformation test.

	Unstandardized coefficient	95% CI lower bound	95% CI upper bound	Standardized coefficient	*p*
**Gender**					
Male	Ref.				
Female	0.182	0.069	0.295	0.058	0.002
**Age**					
18–29	Ref.				
30–39	−0.012	−0.167	0.143	−0.003	0.879
40–49	−0.030	−0.189	0.129	−0.008	0.712
50^+^	−0.025	−0.217	0.167	−0.005	0.799
**Education level**					
High school or less	Ref.				
Associate degree	0.241	0.080	0.401	0.06	0.003
College graduate	0.256	0.107	0.404	0.076	<0.001
Postgraduate	0.417	0.163	0.671	0.062	0.001
**Monthly income**					
¥0–1,000	Ref.				
¥1,001–5,000	−0.014	−0.191	0.163	−0.004	0.879
¥5,001–10,000	0.203	0.013	0.394	0.057	0.037
¥10,001^+^	0.152	−0.072	0.377	0.034	0.183
**Health status**					
Excellent	Ref.				
Good	0.163	−0.051	0.377	0.007	0.136
Fair or poor	0.029	−0.104	0.162	0.025	0.665
Exposure to official sources^a^	−0.049	−0.138	0.039	−0.026	0.277
Exposure informal sources^b^	−0.044	−0.146	0.059	−0.021	0.404
Trust in official sources^a^	0.337	0.221	0.453	0.141	<0.001
Trust in informal sources^b^	−0.125	−0.220	−0.030	−0.061	0.010
Perceived information quality	0.200	0.112	0.288	0.095	<0.001
Surge areas vs. non-surge areas	0.079	−0.032	0.190	−0.026	0.162
Adj. *R*^2^	0.036				

a Official sources: traditional media, news media sites or apps, work unit/school, health resources and community.

b Informal sources: search engines, social media, short video platforms and interpersonal sources.

As for demographic factors and health status, female participants scored higher than male participants (*β* = 0.052, *p* = 0.002). Higher education was in general positively related to the total score. Compared to those with a high school or lower education, each of the higher education level was associated with stronger performance on the misinformation test: associate degree (*β* = 0.082, *p* = 0.003), college graduate (*β* = 0.076, *p* < 0.001), postgraduate (*β* = 0.130, *p* = 0.001). In terms of monthly income, participants who earned 5,000–10,000 yuan per month scored higher than those earning 1,000 yuan or less (*β* = 0.057, *p* = 0.037). Age and health status were unrelated to the total score. There was also no difference between surge and non-surge areas in this model.

## 5. Discussion

This study examined Chinese residents’ ability to correctly identify misinformation about COVID-19 vaccines during the first outbreak caused by the Delta variant in China in 2021. We analyzed how participants from surge vs. non-surge areas performed on a misinformation identification test, in terms of both item-specific performance and overall performance across the 10-item test. We also examined the relationships between misinformation identification and sociodemographic characteristics, perceived health status, and information-related factors.

### 5.1. Misinformation identification

To assess the sample’s ability to identify COVID-19 vaccine misinformation, we developed a 10-item test that included the most current and widely circulated inaccurate information, rumors, and conspiracy theories. The overall performance of the sample on the test was adequate, averaging 8 out of 10. However, some misinformation items appeared to be more widely believed than others. Many participants endorsed the ideas that vaccination is unsafe for people with chronic diseases such as hypertension and diabetes, and that the domestic vaccine provides protection against the virus for only 6 months. Participants from surge and non-surge areas performed equally well, as both groups correctly identified most of the misinformation statements as false. However, notable differences were also found with specific misinformation items. Participants from surge areas were more likely to correctly reject the ideas that vaccination is unnecessary as long as one takes precautionary measures, and that one could get the second vaccination shot just ten days after the first one. This indicates that people from surge areas had better knowledge on these issues than those from non-surge areas.

### 5.2. Factors associated with misinformation identification

In our study, neither exposure to official sources nor exposure to informal sources was associated with the total number of correct responses in the misinformation identification task. Exposure to informal sources was also unrelated to performance on specific misinformation items. However, exposure to official sources was a significant predictor of correct responses to several specific misinformation items. It positively predicted correct responses on items about the vaccines being unsafe for people over 60 or with chronic diseases. On the other hand, it also negatively predicted correct responses on items about not needing vaccination as long as precautionary measures are taken or when other people around oneself have already been vaccinated. This latter finding is somewhat different from previous studies that found mainstream media and government sources to have consistently positive impact on knowledge and beliefs (25, 31). One possibility might be that China’s success in containing the epidemic has resulted in complacency among the public. At the time of the study, the initial national epidemic was already well under control. Although regional outbreaks still happened occasionally, they were relatively small in scale and were often stamped out quickly with the government’s swift action. Therefore, although exposure to official sources improved the public’s vaccination knowledge in some regards, it might have also lowered the perceived importance of and need for vaccination as a result of consistently positive coverage during the pandemic.

In our study, trust in official sources was positively associated with correct responses on eight misinformation items, and trust in informal sources was negatively associated with correct responses on five items. The same pattern of associations was also observed with the total score of misinformation identification. These findings are consistent with previous studies (25, 46). It appears that, in China, trust in official sources can contribute to the public’s ability to distinguish false from factual information, while trust in informal sources can lead to greater belief in misinformation. Since trust is a precondition for acceptance, these findings further suggest that adopting scientific and factual information from official sources may improve individuals’ vaccine knowledge and intention, while believing in rumors and conspiracy theories circulating on informal and online platforms can contribute to vaccine hesitancy (8).

Consistent with our findings on trust, we found that perceived information quality was also a positive correlate of misinformation identification. When people perceived the COVID vaccine information they received to be of higher quality, their ability to discern false information also improved. Granted, perceived information quality is not the same as actual information quality. But there is reason to believe that perceptions of information quality are driven at least in part by the actual quality of the information people receive through sources of their choice. This suggests that a critical strategy to fight against misinformation about COVID vaccines is to enhance the general quality of the information available to the public. While this may sound like a commonsensical idea, its importance cannot be overestimated. After all, the “infodemic” is all about competition between different kinds of information. The more accurate information is out there, the less the room and opportunities for misinformation to take root. Moreover, equipped with accurate information and sound knowledge, people will also be better able to fend off misinformation when under assault and maintain their ability to make truly informed decisions about their vaccination and other self-protective measures.

The pattern of associations between sociodemographic factors and misinformation identification was complex in our data, demonstrating uneven vulnerability to the influence of misinformation across Chinese society. Certain groups of people, particularly women and those with lower education levels and lower income, appeared to be particularly susceptible to misinformation. These findings point to a critical need for targeted dissemination of high-quality information among these vulnerable groups (52).

### 5.3. Information sources: surge vs. non-surge areas

During times of uncertainty like a disease outbreak, people rely heavily on media and interpersonal sources to appraise personal and collective risk and to inform decision making. In this study, exposure to official and informal sources of COVID vaccine information was at similar levels for participants from surge and non-surge areas. This indicates that personal proximity to the outbreak did not make a significant difference in how much people used various types of sources. From another angle, this suggests that both official and informal sources were important information providers regardless of local outbreak status. In China, official sources such as government-owned media and professional health organizations generally provide more reliable and accurate information (61), but their information delivery is not always timely due to policy and procedural constraints. By contrast, informal sources such as social media and personal networks are easier and faster to access, even though the quality of the information disseminated through these channels may not always be unimpeachable. It appears that, during the outbreak, each type of information sources had some advantages to offer and people in both surge and non-surge areas had settled on a balanced diet to fulfill their information needs.

It is important to note that source usage and trust are two different matters in health information acquisition. Our results showed that trust in official sources was higher than that in informal sources in both surge and non-surge areas. This is unsurprising and consistent with previous evidence that people generally put more trust in professional and authoritative sources than in lay media or interpersonal sources (62). Our result also showed that trust in informal sources in surge areas was significantly lower than in non-surge areas. When the outbreak happened, people in surge areas were facing a much more urgent need to make vaccination decisions to protect themselves. In other words, perceived risk and urgency might have affected trust in information sources. When facing high risk and the need to make an important self-protective decision, people relied more on information that they believed to be accurate, and placed less trust in unverified information sources.

### 5.4. Practical and theoretical implications

This study yields several practical implications for COVID vaccine promotion in China and globally. First, we suggest information providers to promote accurate knowledge and reducing public uncertainty about the COVID-19 vaccines (36). Both official and informal sources, should strive to enhance their information quality and reduce, if not eliminate, the circulation of misinformation on their platforms. Second, our study reveals that trust is a more critical factor in the public’s ability to recognize and fend off misinformation than simple exposure. Intervention efforts should look to exploit the existing trust structure in the informational environment to boost the impact of their messages. There should also be efforts to build and maintain trust in intervention-owned information outlets, such as campaign websites, to ensure that the public can access and utilize accurate information about the COVID-19 vaccines without undue concerns. Third, health promotion should pay more attention to the role of media use and the public’s media literacy. The findings in this study indicate that helping individuals to identify credible information sources and scientific facts in a complex media environment is crucial for vaccination programs. Besides developing intervention-specific information outlets, we encourage health promotion to be more active on media platforms commonly used by the public and ensure that reliable health information can reach the target audiences. While engaging social media and/or community networks, care should be taken to address the potential muddling of irrelevant and contradictory misinformation circulating in the same spheres. Finally, at times of regional (or larger-scale) outbreak, heavier reliance on high trust sources should prove most beneficial in raising awareness, keeping communities informed, weeding out misinformation, and mobilize appropriate actions such as getting vaccination.

Recent research acknowledges the increasing significance of information-related factors in health issues (63, 64). Various theories, such as the comprehensive model of information seeking and theory of motivated information management, have emerged to illuminate factors driving information behaviors (65, 66). Our work indicates that individuals’ trust in professional sources and perceived information quality are essential to individuals’ ability to resist false messages. To the extent that misinformation identification is an increasingly important form of information management in the current “infodemic” age, findings from this study should have much to contribute to the future development of information management theories. We encourage future research to look into this possibility.

### 5.5. Limitations

Limitations of the current data need to be considered. First, due to strict regulations during the pandemic, sampling for this study was not probability-based. We noticed that the ratios of female and higher education participants were relatively high in this study, potentially a result of selection bias. Second, all data collected in this study were self-reported. In particular, the measures of information quality and frequency of exposure were based on individual assessments and may contain bias. Third, the surge and non-surge areas examined in this study have important socio-economic differences. Although we controlled for a number of socio-economic factors in the main analyses, our results may still be confounded by unobserved differences between the two regions. Finally, the current data are cross-sectional, thus unable to speak to the causal order of the observed relationships.

## 6. Conclusion

This study examined the public’s ability to identify COVID vaccine misinformation in two provinces in China, and investigated the relationships between such ability and a range of sociodemographic, information, and geographic risk variables. We found that trust in information sources was a strong predictor of the public’s ability to identify misinformation and the nature of this relationship varied between official and informal information sources. We also found that perceived information quality mattered in misinformation identification and certain population segments were at greater risk of being misled by false COVID vaccine information. These findings provide useful insights for the continued efforts to promote COVID-19 and other vaccinations in China.

## Data availability statement

The raw data supporting the conclusions of this article will be made available by the authors, without undue reservation.

## Ethics statement

The studies involving humans were approved by the institutional review board of the Social Science, Jinan University. The studies were conducted in accordance with the local legislation and institutional requirements. The ethics committee/institutional review board waived the requirement of written informed consent for participation from the participants or the participants’ legal guardians/next of kin because the survey was conducted online during the pandemic and could not provide written informed consent.

## Author contributions

JL: Conceptualization, Data curation, Funding acquisition, Investigation, Methodology, Project administration, Resources, Supervision, Validation, Writing – original draft, Writing – review & editing. YC: Formal analysis, Investigation, Project administration, Writing – original draft. XZ: Formal analysis, Methodology, Supervision, Writing – original draft, Writing – review & editing. XY: Data curation, Formal analysis, Project administration, Writing – original draft. FW: Writing – review & editing, Conceptualization, Data curation, Funding acquisition, Investigation, Methodology, Project administration, Resources, Supervision, Validation, Visualization, Writing – original draft.

## Footnotes

^1^fact.qq.cn
